# CCL4 Inhibition in Atherosclerosis: Effects on Plaque Stability, Endothelial Cell Adhesiveness, and Macrophages Activation

**DOI:** 10.3390/ijms21186567

**Published:** 2020-09-08

**Authors:** Ting-Ting Chang, Hsin-Ying Yang, Ching Chen, Jaw-Wen Chen

**Affiliations:** 1 Department and Institute of Pharmacology, School of Medicine, National Yang-Ming University, Taipei 11221, Taiwan; tf0619@yahoo.com.tw (T.-T.C.); shin13573@gmail.com (H.-Y.Y.); j463721092@gmail.com (C.C.); 2 Healthcare and Services Center, Taipei Veterans General Hospital, Taipei 11217, Taiwan; 3 Cardiovascular Research Center, National Yang-Ming University, Taipei 11221, Taiwan; 4 Department of Medicine, Taipei Veterans General Hospital, Taipei 11217, Taiwan

**Keywords:** atheroma, atherogenesis, atherosclerosis, CCL4, inflammation, adhesion molecule

## Abstract

Atherosclerosis is an arterial inflammatory disease. The circulating level of the C-C chemokine ligand (CCL4) is increased in atherosclerotic patients. This study aimed to investigate whether CCL4 inhibition could retard the progression of atherosclerosis. In ApoE knockout mice, CCL4 antibody treatment reduced circulating interleukin-6 (IL-6) and tumor necrosis factor (TNF)-α levels and improved lipid profiles accompanied with upregulation of the liver X receptor. CCL4 inhibition reduced the atheroma areas and modified the progression of atheroma plaques, which consisted of a thicker fibrous cap with a reduced macrophage content and lower matrix metalloproteinase-2 and -9 expressions, suggesting the stabilization of atheroma plaques. Human coronary endothelial cells (HCAECs) and macrophages were stimulated with TNF-α or oxidized LDL (ox-LDL). The induced expression of E-selectin, vascular cell adhesion molecule-1 (VCAM-1), and intercellular adhesion molecule-1 (ICAM-1) were attenuated by the CCL4 antibody or CCL4 si-RNA. CCL4 inhibition reduced the adhesiveness of HCAECs, which is an early sign of atherogenesis. CCL4 blockade reduced the activity of metalloproteinase-2 and -9 and the production of TNF-α and IL-6 in stimulated macrophages. The effects of CCL4 inhibition on down-regulating adhesion and inflammation proteins were obtained through the nuclear factor kappa B (NFκB) signaling pathway. The direct inhibition of CCL4 stabilized atheroma and reduced endothelial and macrophage activation. CCL4 may be a novel therapeutic target for modulating atherosclerosis.

## 1. Introduction

Atherosclerosis is a chronic inflammatory disorder of the arteries that leads to cardiovascular morbidity and mortality [1]. Inflammatory cytokines and chemokines play important roles in the pathogenesis and complications of atherosclerosis [2]. Endothelial dysfunction caused by various risk factors, including hyperglycemia, hypertension, low-density lipoprotein (LDL), and other factors, is regarded as the key mechanism for atherogenesis [3]. Circulating LDL can enter the subendothelial layer, where it may be oxidized to oxidized LDL (ox-LDL), as one of the key components of atheroma. Upon stimulation, endothelial cells, together with other vascular cells, may produce various inflammatory mediators, including adhesion molecules and cytokines, such as tumor necrosis factor (TNF)-α, interleukin-1, and interleukin-6. These inflammatory mediators can promote the endothelial adhesion of circulating leukocytes, direct the migration of bound leucocytes into the intima, mature the monocytes into macrophages, and enhance the lipid uptake of macrophages to form the lipid core of atheroma plaques [4]. Importantly, atheroma plaques with a thin fibrous cap, a large necrotic core, and a high content of leucocytes are more inflammatory and vulnerable to rupture, suggesting a high-risk phenotype for acute cardiovascular events [5]. To prevent potential clinical events, it was suggested that a novel anti-inflammatory strategy to stabilize atheroma plaques be identified [6].

C-C chemokine ligand (CCL) 4, one of the ligands of C-C chemokine receptor (CCR) type 5, is related to atherosclerosis [6]. A naturally truncated CCL4, lacking the two NH_2_-terminal amino acids, can also signal through CCR1 and CCR2b [7,8,9]. Secreted by various vascular and blood cells, such as activated leucocytes, lymphocytes, vascular endothelial cells, and pulmonary vascular smooth muscle cells [10], CCL4 is a chemoattractant for the CD4+CD25+ T cell population. T cell adaptive immunity may be involved in vascular inflammation in atherosclerosis, as atherosclerosis can be modulated by specific immune responses against plaque antigens such as ox-LDL [11]. CCL4 can induce reactive oxygen species and activate the in vitro adhesion of THP-1 cells, human monocytic cells, to human endothelial cells [12]. CCL4 is expressed in the infarcted mouse myocardium [13]. Clinically, circulating CCL4 levels are increased in patients with atherosclerosis [14]. CCL4 can be detected in T-cells, smooth muscle cells, and macrophages in atherosclerotic plaques [15,16,17], and further upregulated in vulnerable plaques [16]. Taken together, these observations suggest the potential involvement of CCL4 in atherosclerosis. However, the role of CCL4 has not been well-defined in atherosclerosis in vivo [6]. To address this issue, the current study investigated whether the direct inhibition of CCL4 by a specific antibody could retard the progression of atherosclerosis and promote the stabilization of atheroma plaques in vivo and reduce TNF-α-induced endothelial adhesiveness to monocytic cells in vitro. Our findings may help to clarify whether CCL4 could be a potential anti-inflammatory target for atherosclerosis.

## 2. Results

### 2.1. Direct Inhibition of CCL4 Attenuated Inflammatory Cytokines in Atherosclerotic Mice

**Figure 1:**
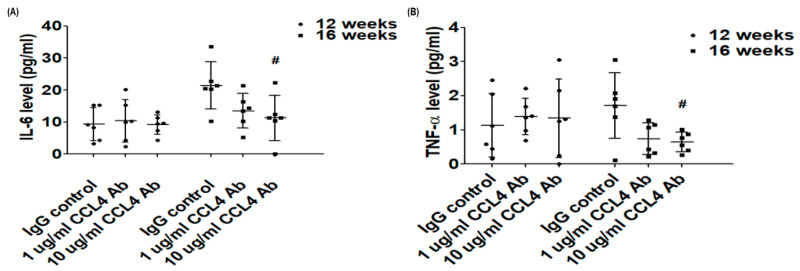

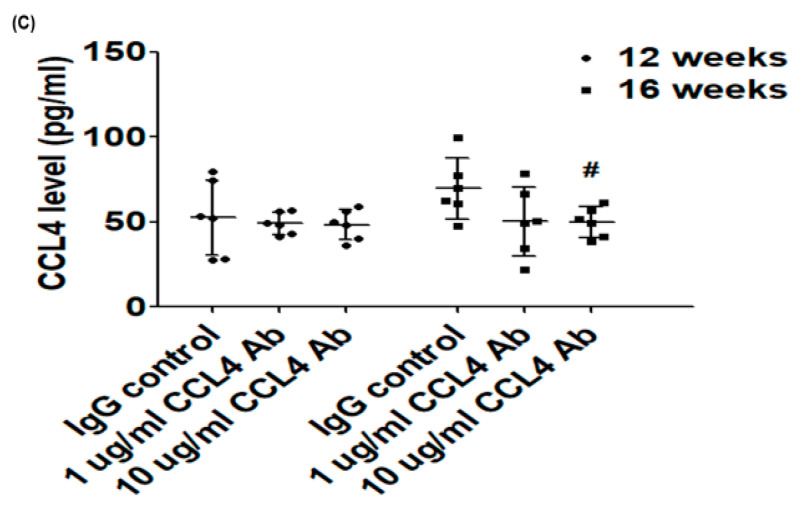
The effects of C-C chemokine motif ligand (CCL4) antibody treatment on cytokines. Serum levels of interleukin (IL)-6 (**A**), tumor necrosis factor (TNF)-α (**B**), and CCL4 (**C**) (*n* = 6 in each group). # *p* < 0.05 compared with the IgG_2A_ isotype control group.

Serum levels of IL-6 and TNF-α were reduced in the CCL4 antibody-treated groups compared to the IgG_2A_ control group (Figure 1A,B). Circulating CCL4 levels were elevated in the IgG_2A_ control group, but were maintained in the CCL4 antibody-treated groups (Figure 1C). These data indicated that the direct inhibition of CCL4 with CCL4 antibodies could efficiently attenuate circulating CCL4 levels and abolish the increase in circulating inflammatory cytokines, along with atherosclerosis progression.

### 2.2. Direct Inhibition of CCL4 Benefited Metabolic Parameters Might Be through the Upregulation of Liver X Receptors (LXRs) in Atherosclerotic Mice

**Figure 2:**
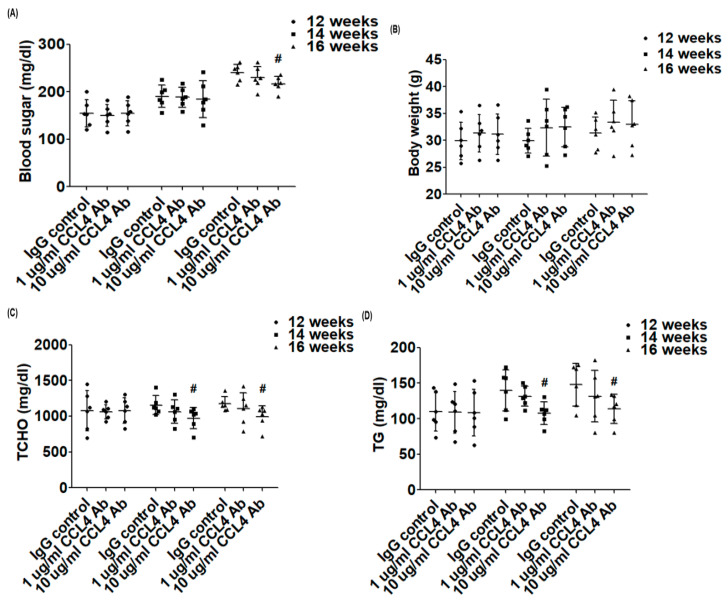

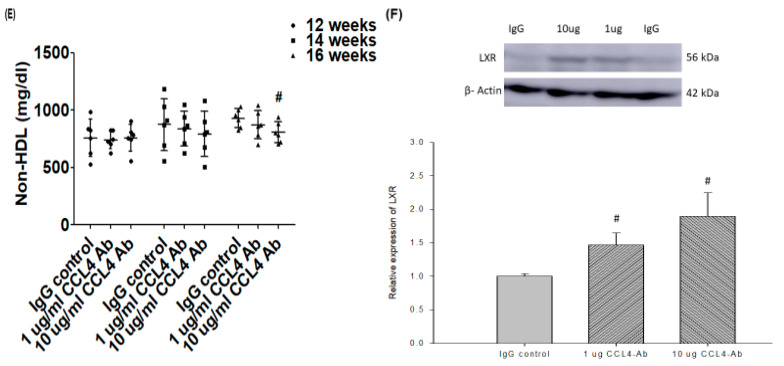
The effects of CCL4 antibodies on metabolic parameters. Blood glucose levels (**A**), body weights (**B**), total cholesterol levels (**C**), triglyceride levels (**D**), and non-high-density lipoprotein (HDL) levels (**E**, *n* = 6 in each group). Western blots and statistical analyses of liver X receptor (LXR) expression in the liver (*n* = 3; **F**). # *p* < 0.05 compared with the IgG_2A_ isotype control group.

We measured the metabolic parameters in each group of mice. Compared to those in the IgG_2A_ control group, blood sugar levels were decreased with the 10 μg CCL4 antibody treatment for 4 weeks (Figure 2A). There was no significant change in body weight in each group during CCL4 antibody treatment (Figure 2B).

It was previously shown that the proportions of lipids in total cholesterol (~14-folds), particularly in the very low-density lipoprotein + intermediate-density lipoprotein fraction (~30-folds), could be dramatically increased in ApoE KO mice fed a Western-type diet [18]. In the present study, serum levels of total cholesterol (TC) (Figure 2C), triglycerides (TG) (Figure 2D), and non-HDL (Figure 2E) in ApoE KO mice were decreased in the 10 μg CCL4 antibody-treated group compared to the IgG_2A_ control group. CCL4 inhibition significantly increased the LXR expression in liver tissues in ApoE KO mice (Figure 2F). The above data showed that CCL4 inhibition could modify the lipid profile, upregulate LXRs, and attenuate the elevated trend in blood sugar levels in atherosclerotic mice.

### 2.3. Direct Inhibition of CCL4 Attenuated Plaque Development and Reduced Macrophage Infiltration in Atherosclerotic Mice

**Figure 3:**
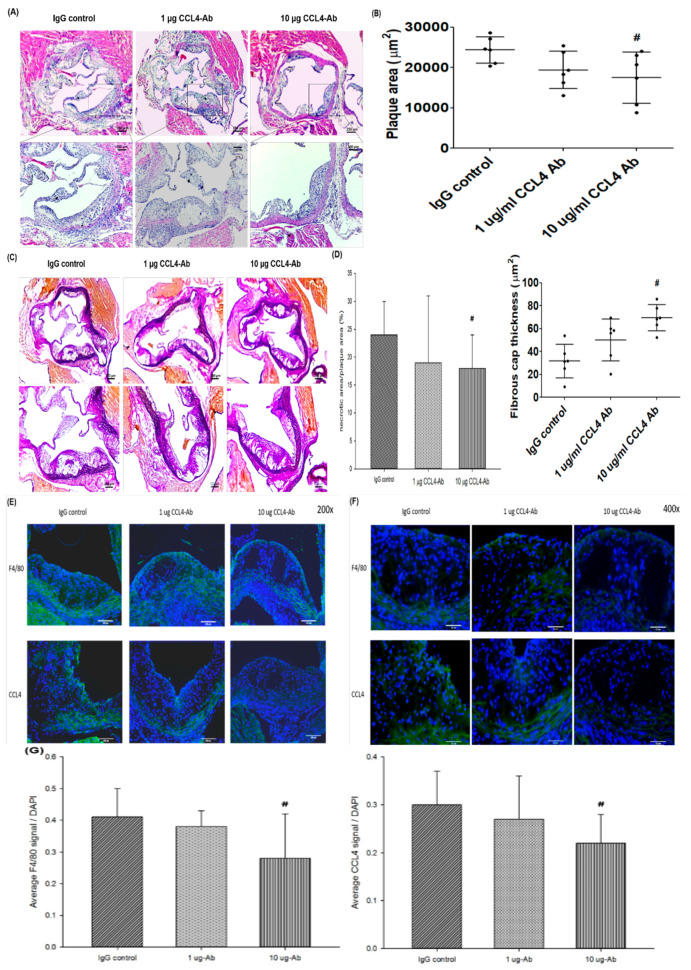
CCL4 antibody treatment reduced the atherosclerotic necrotic area and attenuated macrophage infiltration and CCL4 expressions in plaques. H&E staining of the aorta (**A**; upper: scale bars = 40 µm; lower: scale bars = 100 µm). Quantification of the plaque area (μm^2^) (**B**, *n* = 6 in each group). Elastica van Gieson staining of the aorta (**C**; upper: scale bars = 250 µm; lower: scale bars = 100 µm). Upper: 40× magnification; lower: 100× magnification. Quantification of the necrotic area/plaque area (%) and fibrous cap thickness (μm) (**D**, *n* = 6 in each group). The CCL4 inhibition groups showed reduced numbers of macrophages and CCL4 expression in plaques (**E**, 200× magnification, scale bars = 100 µm; **F**, 400× magnification, scale bars = 50 µm). Quantification of the average F4/80 signal/DAPI and CCL4 signal/DAPI (**G**, *n* = 6 in each group). # *p* < 0.05 compared with the IgG_2A_ isotype control group.

The atherosclerotic lesion area was analyzed and quantified by cross-sectional aortic root staining with H&E staining. The atherosclerotic lesion areas were significantly attenuated by the 10 μg CCL4 antibody treatment for 4 weeks compared to the IgG_2A_ control group (Figure 3A,B). Treatment with CCL4 antibodies increased the fibrous cap thickness in the aorta, and the CCL4 antibody-treated group exhibited smaller necrotic areas compared to the IgG_2A_ control group in ApoE KO mice (Figure 3C,D). Then, we examined the levels of macrophage infiltration into plaques. Levels of the immunoreactive macrophage marker (F4/80) showed that the macrophage content within plaques was decreased in the 10 μg CCL4 antibody-treated group compared to that in the control group. The CCL4 expression in plaques was also reduced in the 10 μg CCL4 antibody-treated group (Figure 3E–G).

In summary, the direct inhibition of CCL4 with a specific antibody resulted in plaques that possessed a thick fibrous cap over a small fatty core in ApoE KO mice. The inhibition of CCL4 reduced the macrophage content and aortic CCL4 expression within plaques. These observations suggested that direct CCL4 inhibition could retard plaque progression and modulated inflammation in vivo.

### 2.4. Blockade of CCL4 Reduced MMP2 and MMP9 Expression In Vivo and Their Expression and Activity in Macrophages In Vitro

**Figure 4:**
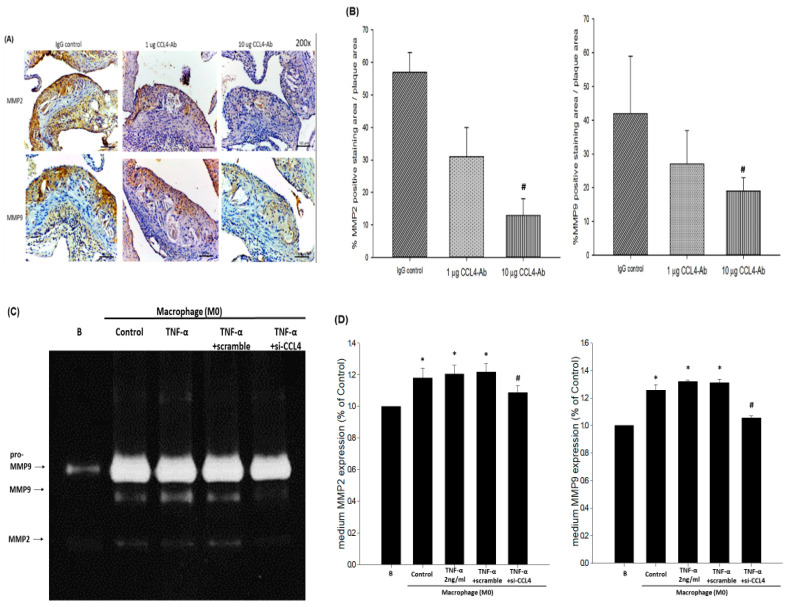

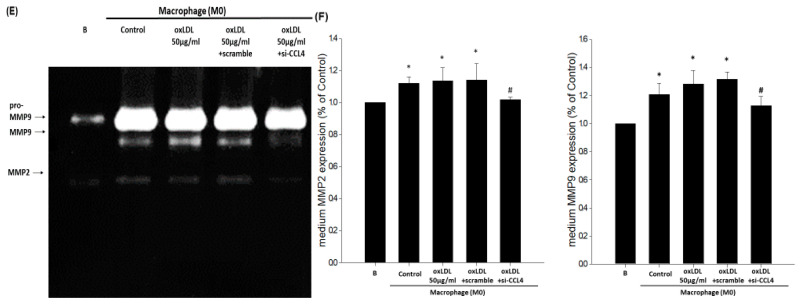
CCL4 blockade down-regulated TNF-α- and oxidized low-density lipoprotein (ox-LDL)-induced matrix metalloproteinase (MMP)2 and MMP9 expression. The CCL4 inhibition groups showed reduced MMP2 and MMP9 expression in plaques (**A**, 200× magnification, scale bars = 100 µm). Quantification of MMP2-positive area/plaque area (%) and MMP9-positive area/plaque area (%) indicating that treatment with 10 μg CCL4 antibody reduced MMP2 and MMP9 expressions in plaques (**B**, *n* = 6 in each group). # *p* < 0.05 compared with the IgG_2A_ isotype control group. MMP9 and MMP2 activities in the culture medium were determined using SDS-PAGE gelatin zymographic analysis (**C**,**E**). Both MMP2 and MMP9 activities were increased after phorbol 12-myristate 13-acetate (PMA) stimulation. The TNF-α- and ox-LDL-induced MMP2 and MMP9 activities were decreased in the CCL4 siRNA-treated group (*n* = 3; **D**,**F**). Basal represented untreated THP-1 cell and control represented THP-1 cell were incubated with PMA to induced M0 phase macrophages. * *p* < 0.05 compared with the basal group. # *p* < 0.05 compared with the TNF-α- or ox-LDL-stimulated group.

The expression of matrix metalloproteinase (MMP)2 and MMP9, which are the mediators contributing to plaque rupture, were decreased in the atheroma plaques of the 10 μg CCL4 antibody-treated animals (Figure 4A,B). To further confirm the resources of MMPs and the effects of endogenous CCL4 inhibition, macrophages were treated with CCL4 siRNA for the in vitro experiment. MMP activity was activated after THP-1 cells were stimulated by after phorbol 12-myristate 13-acetate (PMA) for 48 h. The activity of MMPs were not further enhanced after TNF-α (Figure 4C,D) or ox-LDL (Figure 4E,F) treatments. In the CCL4 siRNA-treated group, the enhanced MMP2 and MMP9 activities were blocked. Taken together, the silencing of endogenous CCL4 could stabilize the atherosclerotic plaques in vivo and reduced MMP2 and MMP9 activities from macrophages in vitro.

### 2.5. Direct Inhibition of CCL4 Decreased TNF-α- and ox-LDL-Induced Endothelial Adhesiveness and Adhesion Molecule Expression

**Figure 5:**
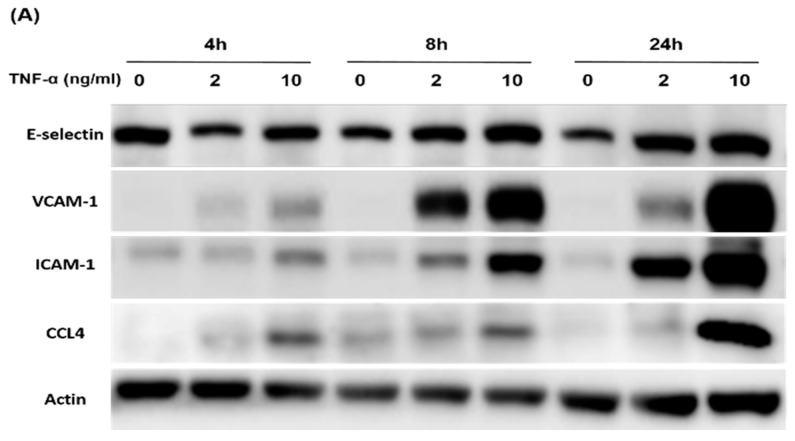

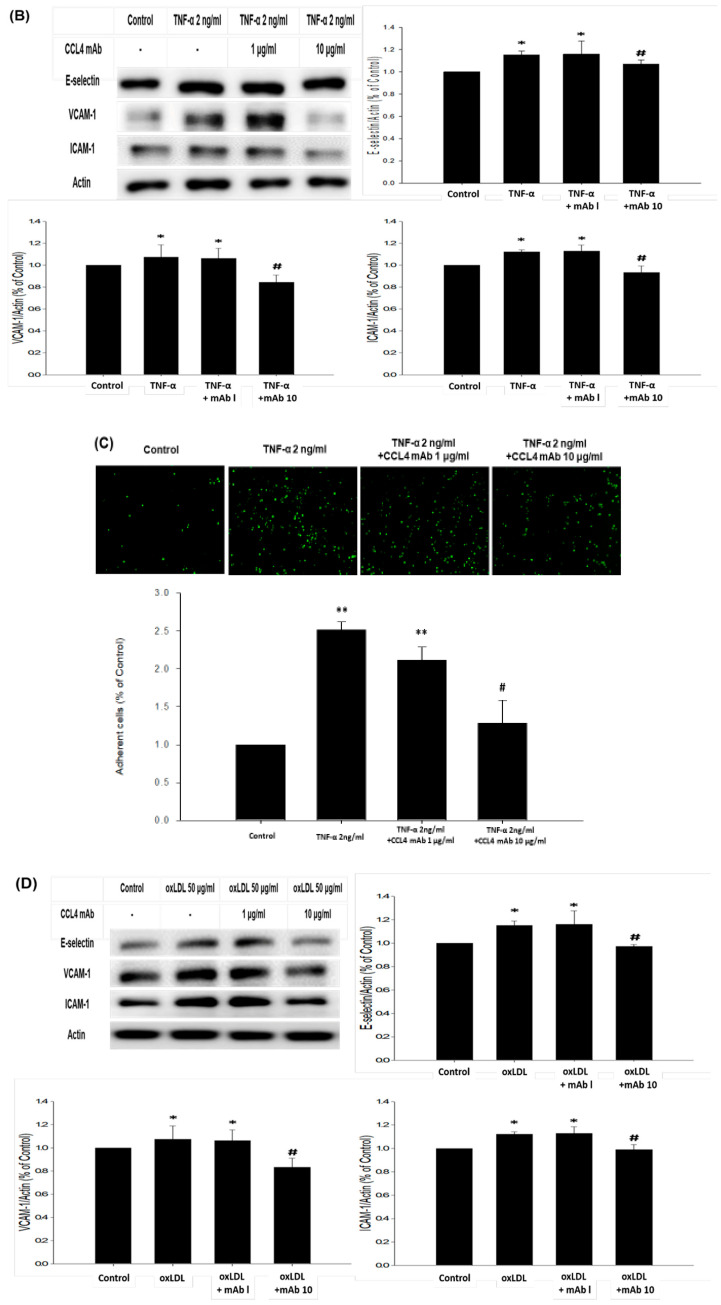

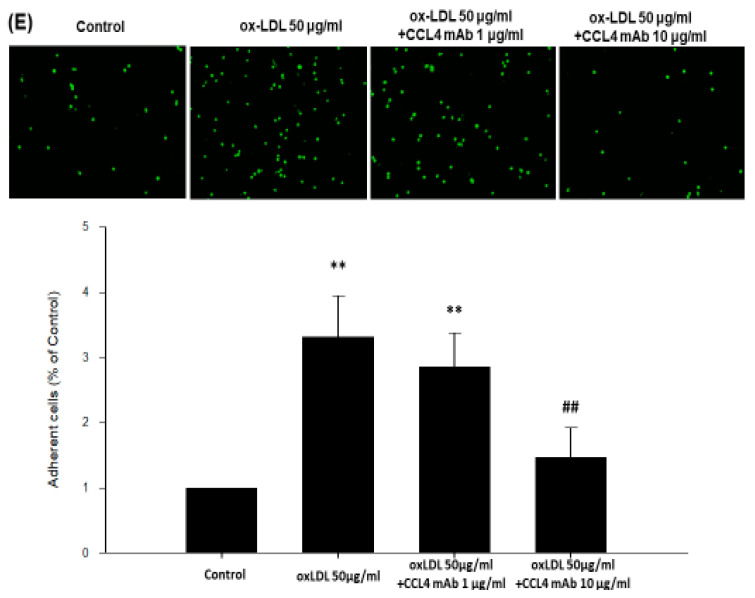
Inhibition of the exogenous CCL4 reduced the TNF-α- and ox-LDL-induced adhesion molecule expression and the adhesiveness of human coronary endothelial cells HCAECs. The time and dose-dependent response of HCAEC after TNF-α stimulations (**A**). Western blots and statistical analyses of E-selectin, vascular cell adhesion molecule-1 (VCAM-1), and intercellular adhesion molecule-1 (ICAM-1) expressions in HCAECs after the stimulation of TNF-α (*n* = 3; **B**) and ox-LDL (*n* = 3; **D**) for 24 h. THP-1 adhesion assay with HCAECs (*n* = 3; **C**,**E**). N represents for the number of independent experiments. * *p* < 0.05, ** *p* < 0.01 compared with the control group. # *p* < 0.05, ## *p* < 0.01 compared with the TNF-α- or ox-LDL-stimulated group.

Both TNF-α and ox-LDL were used as stimulators in the in vitro experiments. Adhesion molecules, such as E-selectin, vascular cell adhesion molecule-1 (VCAM-1), and intercellular adhesion molecule-1 (ICAM-1), as well as CCL4, were induced by TNF-α in a time- and dose-dependent manner in HCAECs (Figure 5A). Accordingly, we tested whether neutralization of the exogenous CCL4 could reduce the expressions of downstream adhesion molecules. The administration of CCL4-specific antibodies decreased TNF-α- (Figure 5B) and ox-LDL-induced (Figure 5D) E-selectin, VCAM-1, and ICAM-1 expression in HCAECs. Furthermore, TNF-α (Figure 5C) and ox-LDL (Figure 5E) induced the adhesiveness of HCAECs to THP-1 monocytic cells, which was reversed by CCL4 antibodies. Taken together, direct CCL4 inhibition with specific antibodies could reduce the TNF-α- and ox-LDL-induced endothelial adhesion of HCAECs in vitro, suggesting a potential role for CCL4 in atherogenesis.

### 2.6. Silencing of Endogenous CCL4 Down-Regulated TNF-α- and ox-LDL-Induced Adhesion and Inflammation Molecules through the NFκB Signaling Pathway

**Figure 6:**
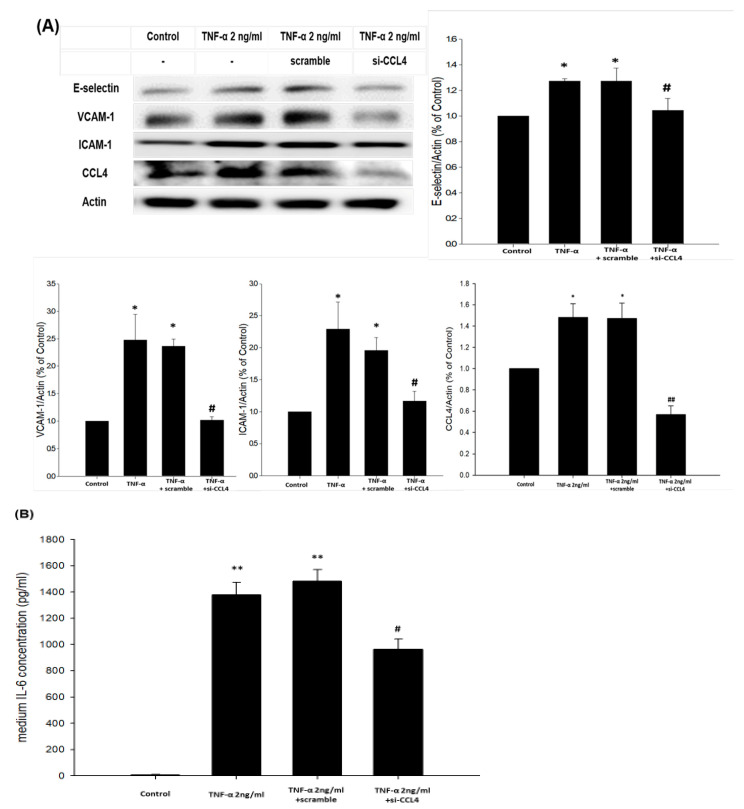

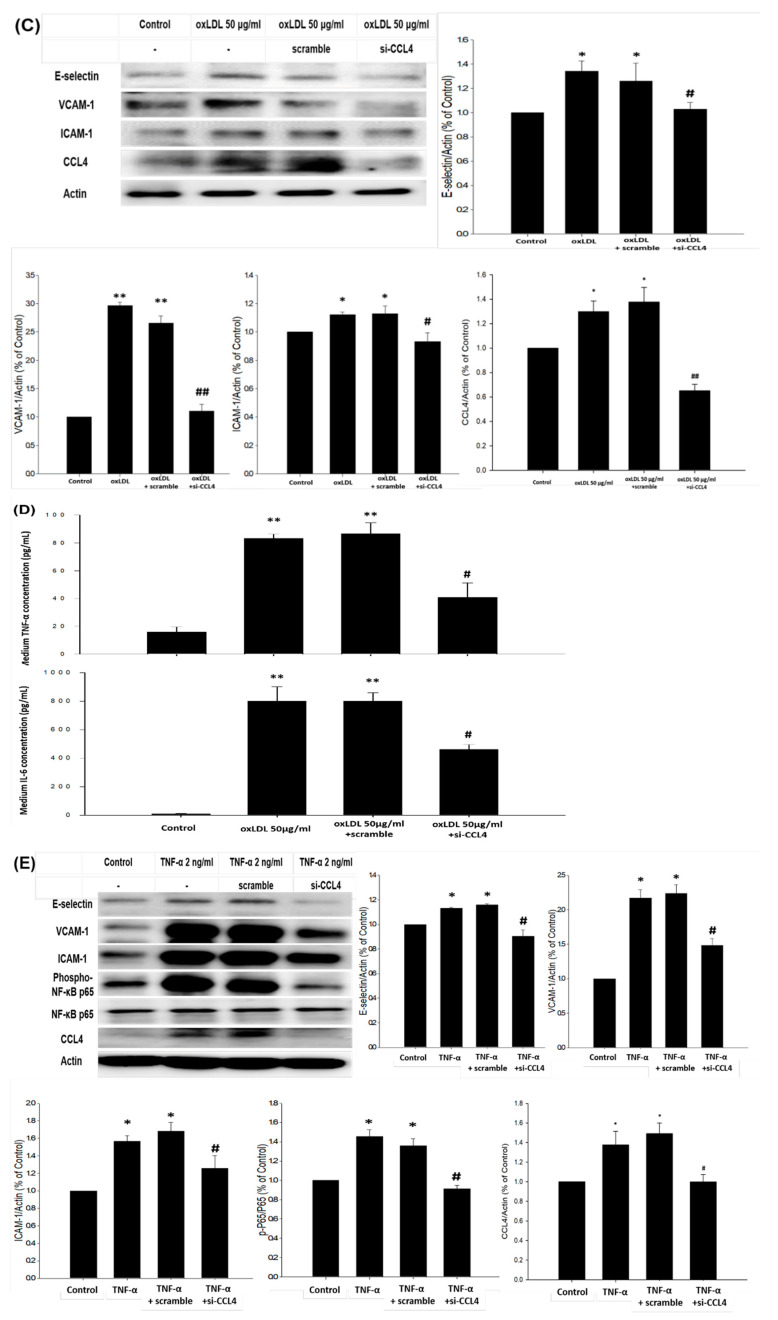

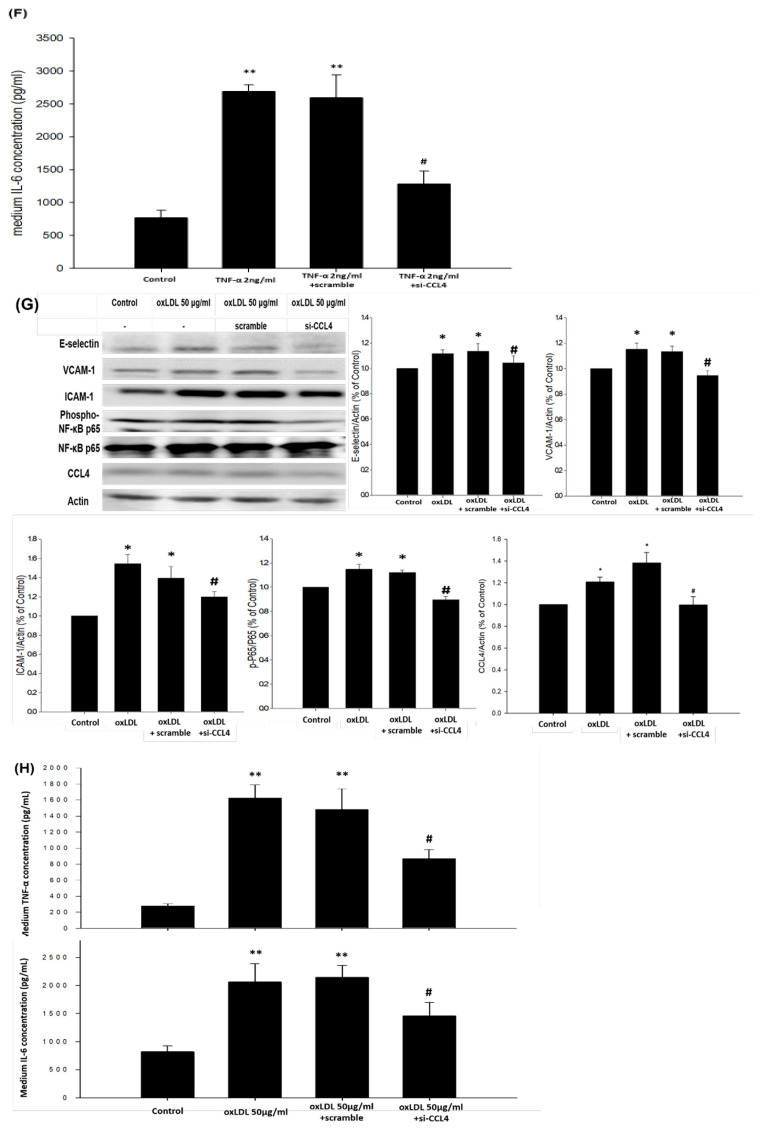
Inhibition of the endogenous CCL4 reduced TNF-α- and ox-LDL-induced adhesion and inflammation protein expressions in HCAECs and macrophages. Western blots and statistical analyses of E-selectin, VCAM-1, and ICAM-1 expressions in HCAECs after the stimulation of TNF-α (*n* = 3; **A**) and ox-LDL (*n* = 3; **C**) for 24 h. TNF-α and IL-6 levels in the culture medium of HCAECs after the stimulation of TNF-α (*n* = 6; **B**) and ox-LDL (*n* = 6; **D**) were determined using ELISA kits. Western blots and statistical analyses of adhesion molecules and the nuclear factor kappa B-p65 (NF-κB) signaling pathway in macrophages after the stimulation of TNF-α (*n* = 3; **E**) and ox-LDL (*n* = 3; **G**) for 24 h. TNF-α and IL-6 levels in the culture medium of macrophages after the stimulation of TNF-α (*n* = 6; **F**) and ox-LDL (*n* = 6; **H**) were determined using ELISA kits. N represents for the number of independent experiments. * *p* < 0.05, ** *p* < 0.01 compared with the control group. # *p* < 0.05, ## *p* < 0.01 compared with the TNF-α- or ox-LDL-stimulated group.

The potential mechanism of the blockade of endogenous CCL4 on TNF-α- and ox-LDL-induced adhesion and inflammation molecules was also investigated. The inhibition of CCL4 with an siRNA reduced the TNF-α- (Figure 6A) and/or ox-LDL-induced (Figure 6C) expression of E-selectin, VCAM-1, and ICAM-1 in HCAECs. Moreover, the IL-6 levels were decreased in the CCL4 siRNA-treated group after the stimulation of TNF-α (Figure 6B); both TNF-α and IL-6 levels were decreased in the CCL4 siRNA-treated group after the stimulation of ox-LDL (Figure 6D). In macrophages, the CCL4 siRNA treatments reduced adhesion molecules after the stimulation of TNF-α (Figure 6E) and ox-LDL (Figure 6G) via down-regulating the phosphorylation of nuclear factor kappa B (NFκB)-p65. The IL-6 levels were decreased in the CCL4 siRNA-treated group after TNF-α treatments (Figure 6F); both TNF-α and IL-6 levels were decreased in the CCL4 siRNA-treated group after ox-LDL treatments (Figure 6H). Although further functional experiments are needed, the above data implied that CCL4 could modulate adhesion and inflammation molecules through the NFκB signaling pathway.

## 3. Discussion

In this study, the direct inhibition of CCL4 with specific antibodies decreased vascular inflammation, reduced the plaque area, and stabilized the vulnerability of atheroma in a mouse model of atherosclerosis in vivo. Furthermore, the inhibition of CCL4 resulted in decreased MMP2 and MMP9 protein expressions and activities in atheroma plaques. In addition, high-dose CCL4 antibody treatments not only improved the metabolic profiles at least partially by upregulating LXR expression, but also reduced the circulating inflammatory cytokines, suggesting systemic anti-inflammatory effects on atherosclerosis. These findings indicate the in vivo role of CCL4 in atherosclerosis and show that the direct inhibition of CCL4 can stabilize atheroma plaques and retard the progression of atherosclerosis. On the other hand, either the blockade of exogenous CCL4 by antibodies or endogenous CCL4 by siRNA could decrease TNF-α- and ox-LDL-induced adhesion and inflammation molecules in HCAECs and the expression of MMP2 and 9 in macrophages. Although further functional experiments should be confirmed, CCL4 could modulate adhesion and inflammation molecules through the NFκB signaling pathway. Taken together, our observations suggested the potential effects of CCL4 in the vulnerability of atheromas and the progression of atherosclerosis, which may be related to its role in the activation of macrophages, as well as endothelial cells. Given the potential contribution of both metabolic risk factors and inflammatory cytokines to the progression of atherosclerosis, the role of CCL4 could be important and complex both in vivo and in vitro. Future studies may be conducted to further elucidate the complex mechanisms of CCL4 and to validate the novel anti-inflammatory strategy targeting CCL4 in other atherosclerosis animal models before potential clinical implications are drawn.

Acute myocardial infarction is one of the most fatal complications of atherosclerosis, which may be due to the rupture of the fibrous caps of plaques [19]. A previous study indicated that a thin, collagen-poor fibrous cap and a macrophage-rich lipid core with increased necrotic areas could destabilize atherosclerotic plaques, which are prone to rupture [20,21]. Both the increased numbers of inflammatory cells [22] and the presence of MMPs [20] are implicated in plaque vulnerability. MMP2 could actively degrade intact fibrillar collagens and weaken plaques. MMP9 could destroy elastin and play a role in outward remodeling and aneurysm formation [23]. In this study, the inhibition of CCL4 decreased the expression of both MMP2 and MMP9, which, together with the increased fibrous cap thickness, decreased necrotic areas, and reduced macrophage content, might further stabilize atheroma plaques in atherosclerotic animals.

The in vivo effects of CCL4 seem complex and crucial for immune responses towards infection and inflammation [24]. CCL4 could activate acute neutrophilic inflammation and induce the synthesis and release of inflammatory cytokines such as IL-1, IL-6, and TNF-α from fibroblasts and macrophages [4]. Previous studies have indicated that CCL3, CCL4, and interferon (IFN)-γ were co-secreted early by natural killer cells and later by CD8+ T/CD4+ Th1 cells. CCL3 and CCL4, together with IFN-γ, could activate macrophages to release nitric oxide and TNF-α [25]. Our findings are in line with previous research and further confirm the systemic effects of CCL4 antibody treatment on circulating IL-6 and TNF-α in in vivo experimental atherosclerosis. Furthermore, both IL-6 and TNF-α were shown to stimulate the production of MMP2 and MMP9 [26,27]. It is thus possible that the inhibition of CCL4 attenuated the tissue expression of MMP9 and MMP2 by modulating circulating IL-6 and TNF-α. In fact, our in vitro data indicated that the silencing of endogenous CCL4 in macrophages could lead to decreased TNF-α- and ox-LDL-stimulated MMP2 and MMP9 activities and reduce IL-6 and TNF-α levels in their culture medium.

In this study, the direct inhibition of CCL4 could decrease circulating IL-6 and TNF-α levels, suggesting the potential modulation of downstream inflammatory cytokines by blocking CCL4 in atherosclerosis in vivo. A pathogenetic mechanism that leads to islet inflammation in type 2 diabetes mellitus begins with chronic metabolic stress, which induces an inflammatory response in pancreatic islets, consisting of an increased production of cytokines and chemokines [28]. CCL4 may be one of the chemokines involved in the development of atherosclerotic diseases via the stimulation of proinflammatory responses and reactive oxygen species [4]. Intracellular stresses such as reactive oxygen species, ceramide, and protein kinase C (PKC) isoforms could modulate insulin signaling via the activation of NFκB and cause insulin resistance [29]. TNF-α and IL-6 have been proposed as a link between obesity and insulin resistance [30]. Chronic exposure to IL-6 could lead to insulin resistance [31]. TNF-α increased free fatty acid production by both adipose tissue and the liver [32]. TNF-α also stimulated lipolysis in human adipose tissue and might increase hepatic cholesterol synthesis by stimulating the activity of β-hydroxy-β-methylglutaryl-CoA (HMG-CoA) reductase [33]. Accordingly, one may speculate that in CCL4 antibody-treated animals, the improvement in blood sugar and lipid profiles might be due to the reduction in inflammatory cytokines such as IL-6 and TNF-α. Furthermore, our findings are also in line with the previous finding that lipopolysaccharide-induced CCL4 production by human monocytes could be positively correlated with serum TC and LDL cholesterol concentrations [34].

LXR regulates lipid metabolism and inflammation by up- and down-regulating its target genes. LXR deletion was linked with an increase in aortic root atherosclerosis and a decrease in plasma TC and TG levels [35]. LXR agonist treatments resulted in a reduction in atherosclerosis in vivo [36]. LXR activation attenuated lipopolysaccharide-induced inflammatory mediators, such as inducible nitric oxide synthase, cyclooxygenase-2, and IL-6, in vitro. Moreover, LXR agonists reduced inflammatory responses in a model of contact dermatitis and decreased inflammatory gene expression in the aortas of atherosclerotic mice in vivo [37]. In this study, we revealed that CCL4 inhibition could increase liver LXR expression accompanied by decreased TC, TG, and non-HDL levels, as well as decreased inflammation markers, such as IL-6 and TNF-α. In summary, our data are in line with those of previous studies and imply that CCL4 might be the key regulator of atherosclerosis through the LXR pathway. Future studies may be conducted to further investigate the impact of inflammation on metabolic and lipid profiles with other anti-inflammatory strategies.

In the current experiments with ApoE KO mice, CCL4 antibodies reduced 10% of the serum cholesterol-reduced plaque area by 28%, which was much more than that which was reported previously. On the other hand, CCL4 was shown to induce the migration of both CD4-positive T cells and memory T cells and to enhance the ability of T cells to bind to an endothelial cell monolayer [38]. Accordingly, it seems that the improvement in lipid profiles, the reduction in inflammatory cytokines, and the inhibition of lymphocyte recruitment might contribute to the beneficial effects of CCL4 on atheroma plaques.

In this study, the beneficial effects of direct CCL4 inhibition on atheroma might be related to the mechanisms mediated by the receptors responsible for CCL4. CCR1, CCR2, and CCR5 have all been suggested as receptors for CCL4. Among them, only CCR5 was related to the progression of atherosclerosis, as some of its ligands, such as CCL3, and CCL4, CCL5, could be detected in atheroma plaques [39]. It was suggested that CCR5 might be important in the later stage of plaque development, rather than in early atherosclerosis [39,40]. However, neither clinical evidence nor data on experimental atherosclerosis are consistent. The role of CCR5 in atherosclerosis remains controversial [6]. Future studies should clarify whether CCR1 and CCR2, in addition to CCR5, could contribute to the beneficial effects of direct CCL4 inhibition on atherosclerosis.

In view of the potential future impact, this study aimed to investigate the in vivo and in vitro role of CCL4 in atherosclerosis and to evaluate the feasibility of the use of CCL4 antibodies to retard the progression of atherosclerosis. However, there were some limitations in this study. First, a previous study indicated that the decreased atherosclerosis development may be associated with the inhibition of Th17-associated cytokines in the spleen in ApoE KO mice [41]. As CCL4 is a chemoattractant for the CD4+CD25+ T cell population, the effects of CCL4 inhibition on T cells, especially Th17 cells, should be further explored. Second, our data showed that the direct CCL4 inhibition could attenuate plaque development with reduced macrophage infiltration and improved the lipid profiles accompanied upregulation of LXR in liver in atherosclerotic mice. In the in vitro part, we demonstrated that blockade of CCL4 could decrease TNF-α- and ox-LDL-induced adhesion and inflammation molecules in endothelial cells and the expression of MMP2 and 9 in macrophages. Nevertheless, whether other types of cells such as lymphocytes or hepatocytes could be also the targets of anti-CCL4 therapy needs further investigations. Third, while the effects of CCL4 antibody may be via its direct inhibition on the circulating CCL4 by neutralization [42], we could not exclude the possibility that CCL4 monoclonal antibody might reduce macrophage or suppress CCL4 release in macrophages. On the other hand, the silencing of endogenous CCL4 in macrophages could result in decreased TNF-α- and ox-LDL-stimulated MMPs activities and reduced inflammatory protein levels. The above data mainly suggest the autocrine mechanism of CCL4. The potential paracrine mechanism of CCL4 in macrophages should be further defined. However, the current study simply showed the direct effects of CCL4 monoclonal antibody on the in vivo atherosclerosis. Given the complex mechanisms that may be involved in the systemic effects of CCL4 and its antibody [6], further experiments are needed to elucidate the molecular mechanisms by which CCL4 antibodies could act on different vascular cells and adipocytes and cold reduce the pro-inflammatory status during atherosclerosis.

## 4. Materials and Methods

### 4.1. In Vivo Study

#### 4.1.1. Animal Model and Study Protocol

The male apolipoprotein E-deficient (ApoE KO) mouse is a well-validated model of atherosclerosis that follows a pattern of progression similar to that of human disease [43]. In the current study, wild-type (WT) and ApoE KO mice on a C57BL/6 background were purchased from Jackson Laboratories. The mice were fed and given water, and were maintained with a 12-h light and dark cycle. After 5 weeks of age, male control C57BL/6 mice were fed a standard chow (as the healthy group), and male ApoE KO mice were fed a Western-type diet (20% fat, 0.15% cholesterol; AIN-76A), for a given period of time (5 to 16 weeks of age). ApoE KO mice fed a Western-type diet received an intraperitoneal injection of anti-CCL4 monoclonal antibodies (#46907) MAB451 (1 or 10 μg per mouse; R&D Systems) or an IgG_2A_ isotype control MAB006 three times per week for 4 weeks (12 to 16 weeks of age). Due to the controlled substance of ketamine and research equipment limitation of inhalation anesthetics, the tribromoethanol (Avertin) dose (240 mg/kg IP) used in this study was selected based on the preparation and dosing recommendations outlined by our Institutional Animal Care and Use Committee. All animal experiments were approved by the Institutional Animal Care and Use Committee (IACUC) of National Yang-Ming University (IACUC number 1050907, on 19 September 2016). All animal experiments conformed to the local approval and all studies of animals were conducted in accordance with the National Institutes of Health Guide for the Care and Use of Laboratory Animals.

#### 4.1.2. Tissue Harvesting

The mice were anaesthetized, and the left ventricles were perfused with PBS (10 mL), with an exit through the severed right femoral artery. The heart and aorta (portion between the heart and the bifurcation of an iliac artery) were harvested, cleaned of adventitial fat, and fixed in 4% paraformaldehyde solution overnight. Aortas were embedded in paraffin.

#### 4.1.3. Histological Staining

Serial sections (8 μm) of the aortic sinus or arch were stained with hematoxylin and eosin to determine the lesion size. Elastica van Gieson staining was used for the visualization of vascular elastic fibers and to determine the collagen content. Quantification analysis was assessed with Motic Images Plus 2.0 software (Plus 2.0, Hong Kong, Kowloon, China).

#### 4.1.4. Immunohistochemical Staining

Immunohistochemical assays were performed with the following primary antibodies: rat F4/80 antibody (Cl-A3-1) NB600-404 (1:50 dilutions; Novus, Centennial, Colorado, USA); rabbit matrix metalloproteinase (MMP) 9 antibody PA5-13199 (1:50 dilution; Thermo Scientific, Waltham, MA, USA); rabbit MMP2 antibody PA1-16667 (4 μg/mL dilution; Thermo Scientific, Waltham, MA, USA); and goat CCL4 antibody (M20) sc-1387 (1:50 dilution; Santa Cruz Biotechnologies, Dallas, TX, USA). Secondary antibodies were purchased from Jackson ImmunoResearch Laboratories, Inc., West Grove, PA, USA. The reaction was visualized by staining with 3,3′-diaminobenzidine (DAB) or fluorescence.

#### 4.1.5. Biochemical Indexes

Serum levels of total cholesterol (TC), triglycerides (TG), and high-density lipoprotein (HDL) after a 4-h fast were determined using an automated clinical chemistry analyzer (FUJI DRI-CHEM 4000i). Blood glucose levels were measured by Optium Xceed.

#### 4.1.6. ELISA

Serum concentrations of interleukin-6, TNF-α, and CCL4 were measured by sandwich ELISA (R&D systems), according to the manufacturer’s instructions.

### 4.2. In Vitro Study

#### 4.2.1. Human Coronary Artery Endothelial Cells (HCAEC) Culture

Primary HCAECs (ScienCell, Carlsbad, CA, USA) were cultured in fibronectin-coated plates with endothelial cell medium containing 5% fetal bovine serum, 1% endothelial cell growth supplement, and 1% penicillin/streptomycin solution at 37 °C in a humidified incubator with an atmosphere of 5% CO_2_.

#### 4.2.2. Adhesion Assay of HCAEC

HCAECs were incubated with TNF-α (2 or 10 ng/mL; PEPROTEC) for 24 h or with TNF-α (2 or 10 ng/mL; PEPROTEC) for 16 h, and then with CCL4 antibodies (1 or 10 μg/mL; R&D Systems) for 8 h, in an atmosphere of 95% air and 5% CO_2_ at 37 °C. The human monocytic THP-1 cell line was originally obtained from the Bioresource Collection and Research Center (BCRC, Taiwan, catalog #60430). Mononuclear cells were labeled with 10 μM BCECF-AM at 37 °C for 1 h in RPMI-1640 medium (Corning, Manassas, VA, USA) and then washed by centrifugation. Confluent HCAECs were incubated with mononuclear cells (5 × 10^5^ cells/mL) at 37 °C for 1 h. Nonadherent mononuclear cells were removed, and plates were gently washed with PBS. The numbers of adherent mononuclear cells were counted in four fields per 200× high-power field per well using a fluorescence microscope (Zeiss, Axiovert 200 M). Six randomly chosen high-power fields were counted per well.

#### 4.2.3. Matrix Metalloproteinase Activity in the Culture Medium of Macrophages

THP-1 cells differentiated with phorbol 12-myristate 13-acetate (PMA) can be used as a model for the function and biology of human macrophages [44]. THP-1 cells were cultured in the presence of PMA (100 nM; Sigma-Aldrich, Darmstadt, Germany) for 48 h. Macrophages were confirmed by the CD11b antibody (NBP2-15774, Novus, Centennial, CO, USA). Macrophages were transfected with ccl4 siRNA (Santa Cruz Biotechnologies, Dallas, TX, USA) using Lipofectamine 2000 (Invitrogen, Waltham, MA, USA) in culture medium. The final concentration of CCL4 siRNA used in in vitro experiments was 30 pmol. Then, cells were treated with TNF-α (2 ng/mL; PEPROTECH, Rehovot, Israel) or ox-LDL (50 μg/mL; KALEN Biomedical, Germantown, MD, USA) for 24 h. Condition medium was collected for analysis.

#### 4.2.4. Gelatin Zymography

MMP9 and MMP2 activities were determined using SDS-PAGE gelatin zymographic analysis. The samples were diluted in sample buffer (2% SDS, 125 mM Tris–HCl, pH 6.8, 10% glycerol, and 0.001% bromophenol blue) and subjected to electrophoresis on 10% SDS-PAGE co-polymerized with gelatin (1%) as the substrate. After electrophoresis, the gel was incubated for 1 h at room temperature in a 2% Triton X-100 solution, washed twice with water, and incubated at 37 °C for 36 h in Tris–HCl buffer, pH 7.4, containing 10 mM CaCl_2_. The gels were stained with 0.05% Coomassie Brilliant Blue R-250, and then destained with 30% methanol and 10% acetic acid. Gelatinolytic activities were detected as unstained bands against the background of Coomassie Blue-stained gelatin. The MMP2 and MMP9 were identified as bands at 72 and 92 kDa, respectively.

#### 4.2.5. Western Blotting

There were three groups of blood vessel tissues. The blood vessels were dissected into two sections for different groups. Different groups of blood vessel tissues and equal amounts of liver tissues were lysed in RIPA lysis buffer (50 mM Tris-HCl pH 7.4, 1% NP-40, 0.5% Na-deoxycholate, 0.1% SDS, 150 mM NaCl, 2 mM EDTA, and 50 mM NaF) and protease inhibitor cocktail (Calbiochem, Darmstadt, Germany), and incubated for 1 h on ice. After centrifugation, the supernatants contained whole cell lysates. Protein concentrations were measured by the BCA assay (Thermo Scientific, Waltham, MA, USA). Equal amounts of HCAEC protein were subjected to SDS-PAGE using 4–12% gradient gels under reducing conditions (Bio-Rad Laboratories, Hercules, CA, USA) and transferred to PVDF membranes (GE Healthcare, Chicago, IL, USA). PVDF membranes were activated by methanol before electroblotting the separated proteins onto the membranes in transfer buffer (25 mM Tris pH 8.8, 192 mM glycine, and 20% methanol). Membranes were probed with monoclonal antibodies against liver X receptor (LXR) (Santa Cruz Biotechnology; sc-271064, Dallas, TX, USA; sc-271064), vascular cell adhesion molecule-1 (VCAM-1; Cell Signaling, Danvers, MA, USA), intercellular adhesion molecule-1 (ICAM-1; Cell Signaling, Danvers, MA, USA), nuclear factor kappa B-p65 (NFκB-p65; BD Biosciences, Franklin Lakes, NJ, USA), phospho-NFκB-p65 (Cell Signaling, Danvers, MA, USA), and β-actin (Chemicon, Tokyo, Japan) at 4 °C overnight. After incubation with secondary antibodies, the bands of probed proteins were visualized by using chemiluminescence detection reagents, according to the manufacturer’s instructions.

#### 4.2.6. Statistical Analysis

The results are given as the means ± standard errors of the mean (SEM). Statistical differences were assessed by one-way analysis of variance (ANOVA) or Student’s *t*-test, followed by post hoc test. A *p*-value of < 0.05 was considered statistically significant.

## 5. Conclusions

The direct inhibition of CCL4 modified metabolic profiles, inhibited vascular inflammation, reduced the plaque area, and promoted a relatively stable plaque phenotype in an experimental model of atherosclerosis in vivo. It could also reduce the activation of macrophages and TNF-α- or ox-LDL-induced adhesion molecules, as well as the endothelial adhesiveness to THP-1, in vitro. Our findings support the role of CCL4 in atherosclerosis and provide a rationale for a novel anti-atherosclerosis strategy targeting CCL4. Given the recent success of the novel anti-inflammatory strategy using anti-IL-1 antibody and others to modify systemic immune response in patients with acute coronary syndrome [45,46,47], further investigations of CCL4 may be interesting in terms of validating the anti-CCL4 strategy for potential clinical use in atherosclerotic diseases.
